# A metabolite of the gut microbiota: a facilitator of chemotherapy efficacy in cancer

**DOI:** 10.1038/s41392-023-01506-4

**Published:** 2023-06-09

**Authors:** Byungsuk Kwon

**Affiliations:** School of Biological Sciences, University of Ulsan, Ulsan, Republic of Korea

**Keywords:** Drug development, Molecular medicine

A recent study has identified a surprising metabolic connection between the gut microbiota and intratumoral neutrophils that impairs the oxidative stress adaptation response to chemotherapy in pancreatic cancer.^1^ This finding suggests that a microbiota metabolite might be used as an additive in improving chemotherapeutic efficacy for cancer patients.

**Fig. 1:**
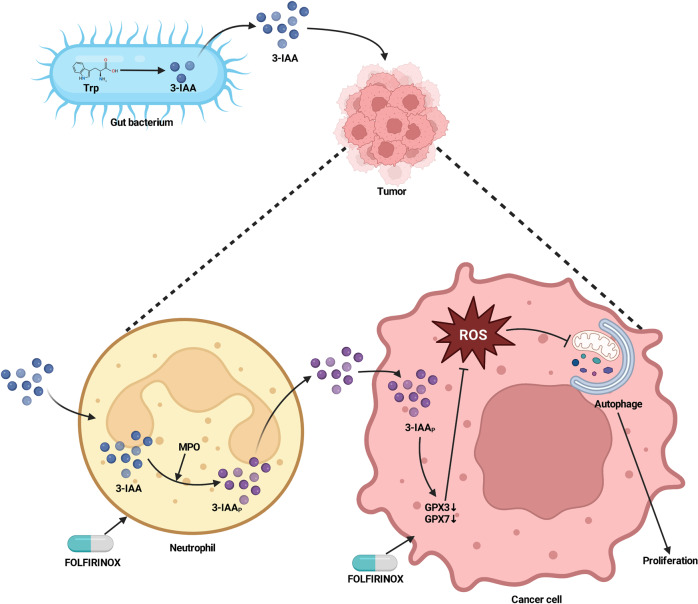
A tryptophan metabolite derived from the gut microbiome accelerates chemotherapy response of pancreatic cancer. Bacteria dwelling in the gut produce indole-3-acetic acid (3-IAA) from uptaken food-derived tryptophan (Trp). 3-IAA translocates to pancreatic ductal adenocarcinoma (PDAC) through the circulation and may be oxidized to toxic molecules (3-IAA_P_) by myeloperoxidase (MPO) and cytotoxic anticancer drugs of 5-fluorouracil, irinotecan and oxaliplantin (FOLFIRINOX) in intratumoral neutrophils. 3-IAA and FOLFIRINOX in turn induce the downregulation of GPX3/7, reactive oxygen species (ROS)-degrading enzymes, and subsequent accumulation of ROS in cancer cells. Finally, increased levels of ROS suppress the autophagy pathway that is critical in cancer cell proliferation (Created in Biorender.com)

Rapid development of high throughput technologies has revolutionized our understanding of human malignancies and shed light on insights into cell-intrinsic and cell-extrinsic factors that drive tumor formation and metastasis, enabling tailoring of therapeutic strategies. Although the cancer genome landscape is predictive of tumor progression and response to therapy, such tumor features can be determined by non-genomic cancer cell state that the tumor microenvironment (TME) dictates. Emerging studies indicate that environmental factors, including the intestinal microbiota, also contribute to therapy. The gut microbiota has been evolved for symbiosis through suppression of the host immunity but its composition can affect cancer therapeutic efficacy in a positive or negative way. Therefore, there is an urgent need to identify gut microbiota-derived metabolites that influence therapy efficacy in responder (R) or non-responder (NR) cancer patients (Fig. 1).

Pancreatic ductal adenocarcinoma (PDAC) is an intractable disease, considering the low 5-year survival. Efforts to find targetable genetic alterations or stratify patients who benefit from immunotherapy have been failed. However, two factors-amounts of high-quality neoantigen-reactive CD8^+^ T cells and a high tumor microbial diversity-determine long-term survival of a small subsets of PDAC patients.^2,3^ Although there is no experimental evidence showing causal association of pre-existing microbes with antitumor immunity in long-term PDAC survivors, existence of neoantigen molecular mimicry with microbial epitopes circumstantially supports the possibility that tumor-dwelling microbes can translocate from the gut and shape the TME favorable for antitumor immunity. Due to difficulties in early detection of PDAC, combinations of cytotoxic chemotherapies remain the mainstay of treatment for patients with advanced and metastatic PDAC. Tintelnot et al. have provided data suggesting that gut microbiota-derived indole-3-acetic acid (3-IAA), a chemical known as an immunosuppressive aryl hydrocarbon receptor (AHR) ligand, unexpectedly augments the therapeutic efficacy of chemotherapy to PDAC patients.^1^

Tintelnot et al. developed the orthotopic, gnotobiotic mouse tumor model where mice were transplanted with the gut microbiome derived from R patients treated with combinations of 5-fluorouracil, irinotecan, oxaliplatin and folinic acid (FOLFIRINOX) or NR patients. They identified a positive correlation between colonization of R microbiota with the responsiveness to FOLFIRINOX in gnotobiotic mice. In their model, transplanted bacteria were poorly established within orthopotic tumor, suggesting that a colonized gut microbial factor(s) that translocated to the tumor site might control the response to chemotherapy. Liquid chromatography-mass spectrometry screening identified 3-IAA as one of the most abundant metabolites in the serum of R patients and R microbiota-colonized gnotobiotic mice. There were not only increased numbers of 3-IAA-producing bacteria in R patients but also correlations between increased serum concentrations of 3-IAA with decreased tumor weight after oral injection of high amounts of tryptophan in R microbiota-colonized gnotobiotic mice. 3-IAA-specific inhibition of tumor growth was experimentally proved such a way that direct oral injection of 3-IAA was sufficient to increase the efficacy of chemotherapy in specific pathogen-free mice. Mechanically, neutrophil myeloperoxidase (MPO) was required for tumor growth inhibition mediated by 3-IAA and FOLFIRINOX. As MPO is known to oxidize 3-IAA into cytotoxic products, Tintelnot et al. investigated in detail how MPO products of 3-IAA oxidation mediate inhibition of tumor growth. They found that 3-IAA-oxidized derivatives, together with FOLFIRINOX, were critical in induction of two main alterations in cancer cells, which rendered them less proliferative and viable. One was accumulation of reactive oxygen species (ROS) that were caused by downregulation of ROS-degrading enzymes glutathione peroxidase 3 and glutathione peroxidase 7 (GPX3 and GPX7). Using knockdown assays of *Gpx3* and *Gpx7* and the ROS inhibitor N-acetylcysteine, Tintelnot et al. demonstrated that accumulation of ROS in pancreatic tumor cells was indispensable for the therapeutic efficacy of FOLFIRINOX. The other was downregulation of the autophagy pathway linked directly to the reduced proliferation of tumor cells. In aggregates, these results suggest that a gut microbiome tryptophan metabolite, in cooperation with chemotherapy, induces the increased oxidative stress and impaired adaptation to such stress, resulting in decreased proliferation of pancreatic cancer cells.

The findings of Tintelnot et al. have important clinical implications, even though several new issues are raised to be answered for. 3-IAA is a promiscuous tryptophan metabolite produced by the gut microbiota and host. Its upstream and downstream products are ligands of AHR that control gut homeostasis and immune regulation. The efficacy of 3-IAA and FOLFIRINOX is independent of AHR of immune and cancer cell origin, suggesting that 3-IAA-oxidized derivatives have anti-proliferative effects on cancer cells in a cell-intrinsic fashion. Nevertheless, it is noteworthy that untreated PDAC tumor is likely to create an immunosuppressive TME enriched with endogenously produced AHR ligands, as human pancreatic adenocarcinoma expresses IL-4-induced-1, an enzyme that has the ability to convert tryptophan to indole-3-pyruvic acid (I3P) and its downstream products, including 3-IAA, indole-3-lactic acid, indole-3-aldehyde (I3A), and kynurenic acid.^4^ Thus, FOLFIRINOX may enable neutrophil MPO to oxidize either gut microbiota- or cancer cell-derived 3-IAA to toxic products. This interpretation may provide an adequate explanation for why 3-IAA-oxidized derivative-mediated antitumor effects override the protumoral function of AHR. It seems that cell death caused by treatment with 3-IAA and FOLFIRINOX is not immunogenic, as T cells are dispensable for the efficacy of 3-IAA and FOLFIRINOX treatment. In aggregates, indole derivatives, including 3-IAA oxidized products and AHR ligands, have no effect on antitumor immunity during chemotherapy^1^ or do not create an immunosuppressive TME. In this aspect, immunotherapy (including AHR antagonists) may have synergy with FOLFIRINOX in treating R patients with PDAC.

A 3-IAA and FOLFIRINOX combination therapy was effective in the treatment of other types of cancer such as colorectal and lung cancer in mice.^1^ In a small PDAC cohort, there was a negative correlation between serum concentrations of 3-IAA and the number of neutrophils during FOLFIRINOX chemotherapy (3-IAA and FOLFIRINOX induces neutrophil necrosis and decreases neutrophil numbers in vivo), while patients with higher serum concentrations of 3-IAA showed a better progression free or overall survival. Accordingly, the direct treatment of 3-IAA might be considerable to improve the outcomes of PDAC patients whose primary therapeutic option is FOLFIRINOX. Another recent study also has discovered a new mechanism of how a gut microbiome-derived tryptophan metabolite called I3A increases antitumor immunity via activation of AHR in CD8^+^ T cells and has synergy with inhibition of immune checkpoints.^5^ Taken together, tryptophan catabolites of the gut microbiota deeply influence tumor growth in a variety of ways, which may depend upon types of cancer and therapy and the composition of the intestinal and tumor microbiome.
